# Post-Progression Survival Is Strongly Associated with Overall Survival in Patients Exhibiting Postoperative Relapse of Non-Small-Cell Lung Cancer Harboring Sensitizing *EGFR* Mutations

**DOI:** 10.3390/medicina57050508

**Published:** 2021-05-19

**Authors:** Hisao Imai, Ryoichi Onozato, Maiko Ginnan, Daijiro Kobayashi, Kyoichi Kaira, Koichi Minato

**Affiliations:** 1 Gunma Prefectural Cancer Center, Division of Respiratory Medicine, Ota, Gunma 373-8550, Japan; kminato@gunma-cc.jp; 2 Comprehensive Cancer Center, International Medical Center, Department of Respiratory Medicine, Saitama Medical University, Hidaka, Saitama 350-1298, Japan; kkaira1970@yahoo.co.jp; 3 Gunma Prefectural Cancer Center, Division of Thoracic Surgery, Ota, Gunma 373-8550, Japan; ronozato@gunma-cc.jp; 4 Gunma Prefectural Cancer Center, Division of Pharmacy, Ota, Gunma 373-8550, Japan; ma-ginnan@gunma-cc.jp; 5 Gunma Prefectural Cancer Center, Division of Radiation Oncology, Ota, Gunma 373-8550, Japan; dkobayashi1988@gunma-cc.jp

**Keywords:** EGFR-TKI, *EGFR* mutation, non-small-cell lung cancer, overall survival, postoperative relapse, post-progression survival, relapse-free survival

## Abstract

*Background and Objective*: Patients with advanced non-small-cell lung cancer (NSCLC) harboring sensitizing epidermal growth factor receptor (*EGFR*) mutations show a good response to EGFR-tyrosine kinase inhibitors (EGFR-TKIs). The subsequent treatments influence the evaluability of the efficacy of front-line therapy on overall survival (OS). Consequently, we evaluated the associations of relapse-free survival (RFS) and post-progression survival (PPS) with OS in patients who exhibited postoperative relapse of *EGFR*-mutated NSCLC. *Materials and Methods*: We analyzed the data of 35 patients with *EGFR*-mutated NSCLC who underwent complete resection between January 2007 and June 2019. The correlations of RFS and PPS with OS were evaluated at the individual patient level. *Results:* Linear regression and Spearman’s rank correlation analyses demonstrated that the PPS highly correlated with OS (*r* = 0.91, *p* < 0.05, *R*^2^ = 0.85), whereas the RFS weakly associated with OS (*r* = 0.36, *p* < 0.05, *R*^2^ = 0.25). Age and performance status at relapse were significantly associated with PPS. *Conclusion*: Overall, PPS was more strongly and significantly associated with OS than RFS. These results suggest that the OS of our cohort may be affected by treatments, besides postoperative relapse. However, larger-scale prospective studies are needed to confirm these results.

## 1. Introduction

Lung cancer is a major reason for cancer-related mortality globally, and non-small-cell lung cancer (NSCLC) accounts for approximately 80% of all lung cancers [1]. For early stage NSCLC, surgical resection is considered the most effective strategy, with the highest potential for improving survival and cure. However, despite complete resection, recurrence and death occur in approximately half of the cases with stage I–IIIA NSCLC [2,3]. It is highly unlikely that postoperative relapse of NSCLC is curable, and the median survival beyond relapse is 8.1–17.7 months [3,4]. An optimal therapeutic strategy for postoperative relapse of NSCLC is expected to alleviate clinical symptoms, maintain quality of life, and slow down disease progression.

With the increasing number of treatment options for NSCLC, the efficacy of front-line therapy on overall survival (OS) might be affected by subsequent treatments [5]. A phase III trial demonstrated that prolonged progression-free survival (PFS) does not always result in prolonged OS of patients with NSCLC [6]. Thus, PFS after first-line therapy is not an ideal alternative endpoint for OS. Instead, post-progression survival (PPS), which is calculated as the difference between OS and PFS, is reportedly highly correlated with OS following first-line therapy with molecular targeted agents, such as EGFR-tyrosine kinase inhibitors (TKIs) [7,8,9]. Previously, we reported that PPS has a stronger significance on OS than PFS in patients with NSCLC harboring sensitizing epidermal growth factor receptor (*EGFR*) mutations treated with first-line EGFR-TKIs. This means that treatment beyond disease progression after front-line treatment may have a significant influence on the OS of patients with NSCLC [10].

The clinical characteristics and prognoses of patients with *EGFR*-mutated NSCLC versus those without *EGFR* mutations have been comprehensively investigated [11,12]. Numerous clinical trials have shown the effectiveness of EGFR-TKIs, such as gefitinib, erlotinib, afatinib, and osimertinib, as a first-line therapy for patients harboring sensitizing *EGFR* mutations [13,14,15,16,17,18,19]. In addition to first-line EGFR-TKIs, other therapeutic choices include platinum-based combination regimens and non-platinum regimens. Approximately 60% of patients who progress after the first-line therapy with a first- or second-generation EGFR-TKI harbor a T790M mutation in *EGFR* [20,21,22,23]. Osimertinib is one of the standard second-line therapy choices for patients with progressive T790M-positive NSCLC following relapse beyond first-line therapy with a first- or second-generation EGFR-TKI [24]. Patients with metastatic T790M-negative NSCLC who progress beyond first-line therapy with a first- or second-generation EGFR-TKI are treated with cytotoxic drugs. However, despite recent large-cohort studies, the clinical and prognostic implications of *EGFR* mutation status in surgically resected lung cancer remain controversial [12,25,26].

It would be interesting to examine the contribution of postoperative relapse treatment to OS at the individual-level. An evaluation of individual patient-level data demonstrated that PPS, and not PFS, is strongly correlated with OS beyond front-line therapy in patients with metastatic NSCLC and small-cell lung cancer (SCLC) [27]. Consequently, continuing therapy beyond postoperative relapse may significantly influence OS. However, the correlation between PPS and OS in postoperative relapse of *EGFR*-mutated NSCLC is currently unclear. Therefore, evaluating individual patient-level data to determine whether relapse-free survival (RFS) and PPS are considerably correlated with OS beyond postoperative relapse is of clinical relevance.

In the current investigation, we evaluated the relationships of RFS and PPS with OS in postoperative relapse patients with NSCLC haboring sensitizing *EGFR* mutations. In addition, we assessed the prognostic significance of a patient’s characteristics for PPS.

## 2. Patients and Methods

### 2.1. Patients

The current study involved patients with postoperative relapse of *EGFR*-mutated NSCLC who underwent a complete resection at Gunma Prefectural Cancer Center between January 2007 and June 2019. The histopathological diagnosis was determined according to the World Health Organization’s classification. The NSCLC stage was determined based on the American Joint Committee on Cancer’s tumor-node-metastasis (TNM) staging system [28]. The inclusion criteria were histologically proven NSCLC, postoperative relapse, and a carcinoma harboring sensitizing *EGFR* mutations (exon 18 *G719X*, exon 19 deletion, exon 21 *L858R*, or exon 21 *L861Q*). In addition, only lobectomies were included, not wedge resection or segmentectomy. Lymph node dissection was also included in this study. On the other hand, the exclusion criteria were operations in other hospitals, incomplete resection, and incomplete data. At postoperative relapse, before treatment, each patient underwent a physical examination, a chest radiograph, a computed tomography of the chest and abdomen, a ^18^F-fluorodeoxyglucose positron emission tomography or bone scintigraphy, and a brain computed tomography or magnetic resonance imaging to determine the disease stage (TNM classification). The medical records of the identified patients were collected and checked. Furthermore, records on patient characteristics, chemotherapeutic regimens, radiotherapy, and subsequent-line treatments (if administered) were collected. First- and higher-line treatments were chosen by the principal medical oncologist and continued until disease progression, unacceptable toxicities, or treatment refusal. Beyond operative recurrence, the patients were permitted to choose any treatment modality following the first-line therapy.

Sensitizing *EGFR* mutations in exons 18–21 were evaluated, as previously demonstrated [29,30], by polymerase chain reaction (PCR) amplification with intron–exon boundary primers. Four sensitizing *EGFR* mutations were identified, of which exon 19 deletion and exon 21 *L858R* were the major sensitizing mutations, whereas exon 18 *G719X* and exon 21 *L861Q* were the minor sensitizing mutations.

This study was approved by the ethics committee of Gunma Prefectural Cancer Center. Owing to the retrospective nature of the study, the requirement for informed consent was waived by the ethics committee. However, the opportunity to refuse participation through an opt-out method was guaranteed.

### 2.2. Treatment Response Assessment

Tumor response was evaluated as the best overall response. Radiological tumor responses were evaluated according to RECIST version 1.1 [31]: disappearance of all target lesions (complete response, CR); a ≥ 30% decrease in the sum of target lesion diameters relative to the baseline level (partial response, PR); a ≥ 20% increase in the sum of target lesion diameters relative to the smallest value during the study (progressive disease, PD); and insufficient shrinkage to qualify as PR and insufficient growth to qualify as PD (stable disease, SD).

### 2.3. Statistical Analysis

We defined RFS as the time from operation to the first instance of relapse or death from any reason. Overall survival was defined as the time from operation until death or censoring at the last consultation. We defined PPS as the time from tumor relapse after operation until death or censoring at the last consultation. Survival curves were generated using the Kaplan–Meier method. Linear regression analyses and Spearman’s rank correlation coefficient were adopted to assess whether RFS and/or PPS were associated with OS. A Cox proportional hazards model with stepwise regression was adopted to identify factors that predicted PPS and to estimate hazard ratios and 95% confidence intervals. Some variables were converted to an appropriate scale unit because the hazard ratio was calculated based on a 1-unit difference. Differences were regarded as statistically significant at a two-tailed *p*-value of ≤0.05. All analyses were conducted using JMP software for Windows, version 11.0 (SAS Institute, Cary, NC, USA).

## 3. Results

### 3.1. Patient Baseline Characteristics and Therapeutic Efficacy

**Figure 1:**
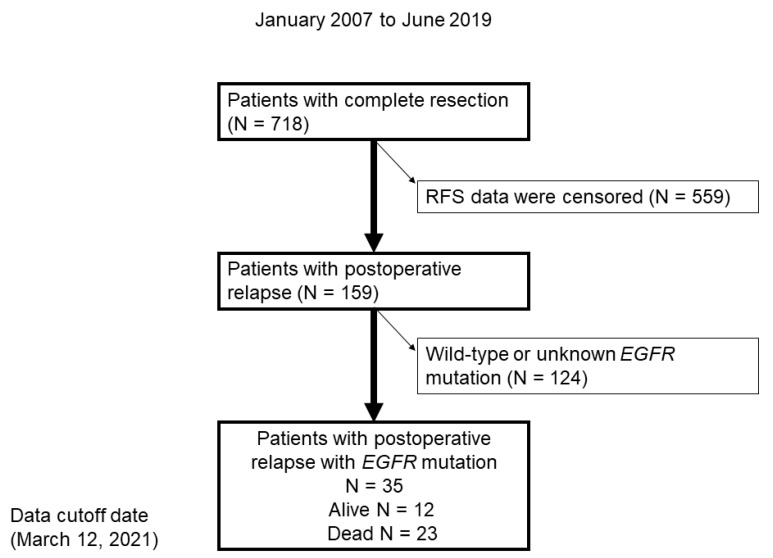
Flow chart demonstrating the identification of patients with postoperative relapse of NSCLC with *EGFR* mutation between January 2007 and June 2019. RFS, relapse-free survival.

In total, 718 patients underwent a complete resection, 159 of whom had postoperative relapse. Subsequently, 124 patients with wild-type *EGFR* or unknown *EGFR* mutation status were excluded (Figure 1). Finally, 35 patients with *EGFR*-positive NSCLC were included in the study.

Of the 35 patients whose data were analyzed in this study, 23 died because of underlying diseases, and 12 are alive. The median follow-up period was 51.6 (range, 11.6–146.5) months. The median patient age was 69 years (range, 44–83 years). The baseline characteristics of the patients are listed in Table 1.

**Figure 2:**
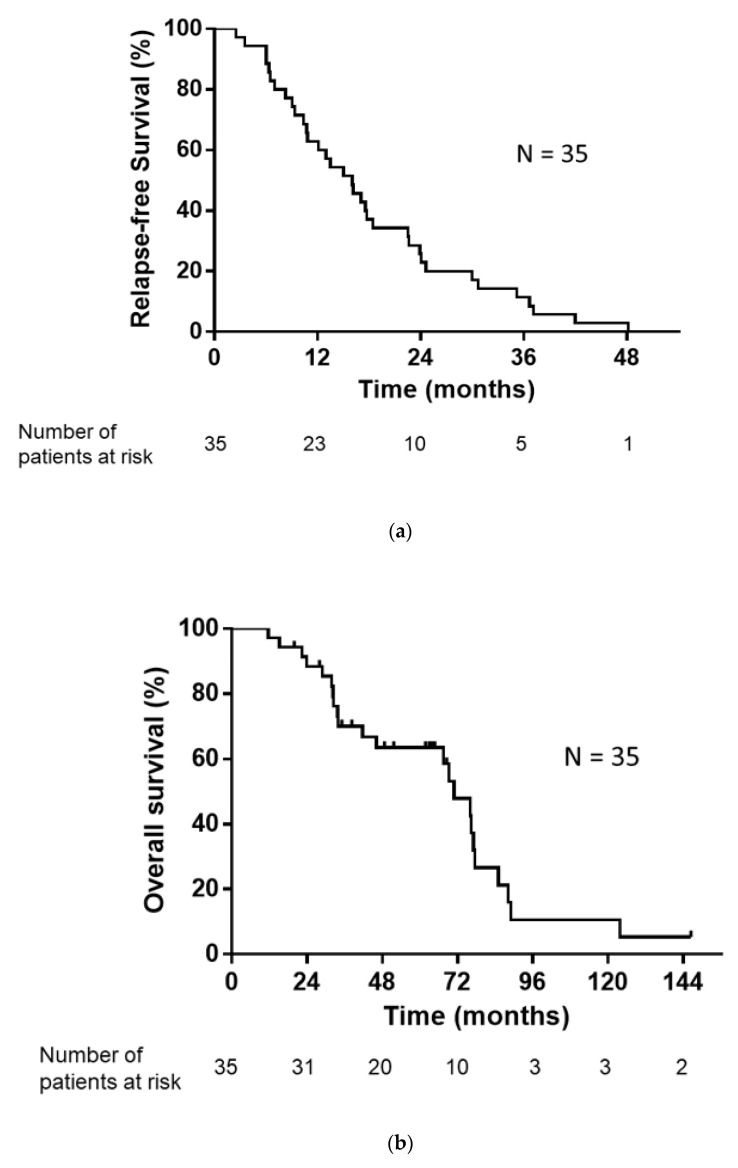
(**a**) Kaplan–Meier plot showing relapse-free survival (RFS). Median RFS: 16.0 months. (**b**) Kaplan–Meier plot showing overall survival (OS). Median OS: 70.9 months.

The median number of regimens after postoperative relapse for the 35 patients was 1 (range, 0–7). The treatments administered following postoperative relapse are listed in Table 2. Of the 35 patients with postoperative relapse, 34 patients (excluding one patient who received only supportive care) received some form of drug therapy or radiotherapy. As an initial treatment following postoperative relapse, 26 patients received EGFR-TKI and three patients received cytotoxic drugs. Five patients underwent definitive thoracic irradiation, including one patient who received concurrent chemoradiotherapy. The median RFS, PPS, and OS were 16.0, 52.2, and 70.9 months, respectively (Figure 2a,b).

### 3.2. Correlations of RFS and PPS with OS

**Figure 3:**
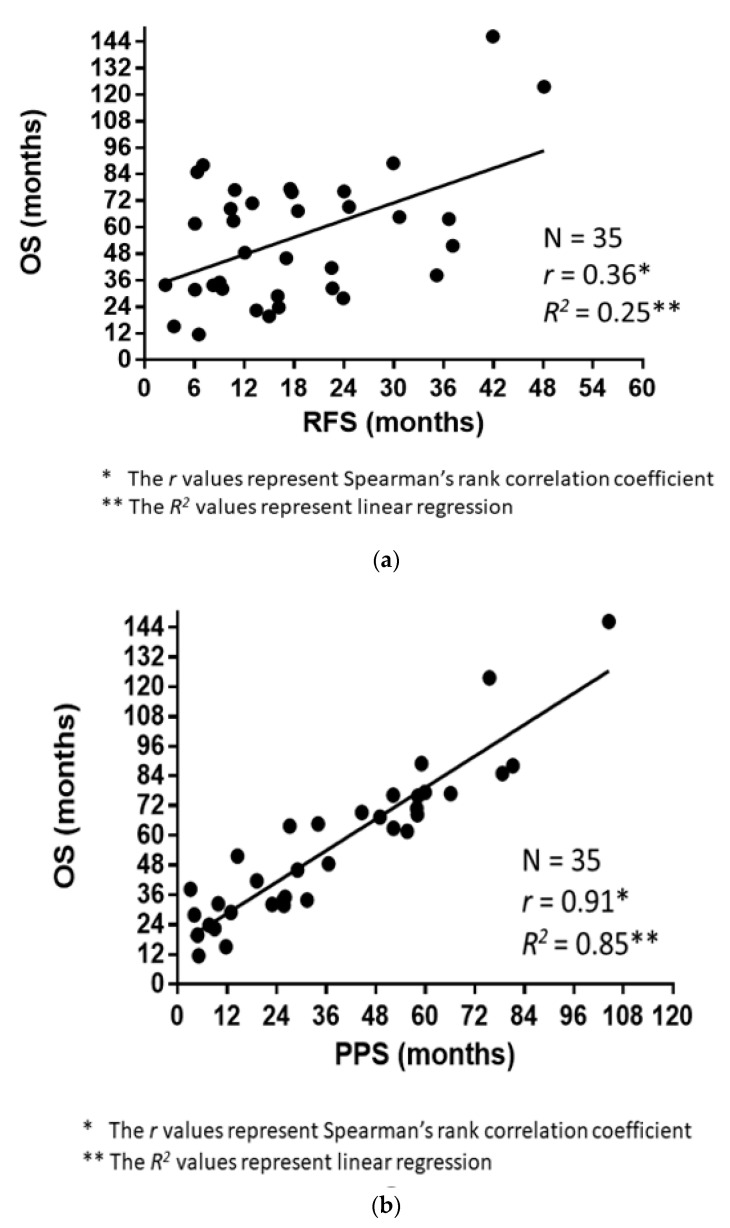
(**a**) Association between the overall survival (OS) and relapse-free survival (RFS). (**b**) Association between the overall survival (OS) and post-progression survival (PPS).

**Figure 4:**
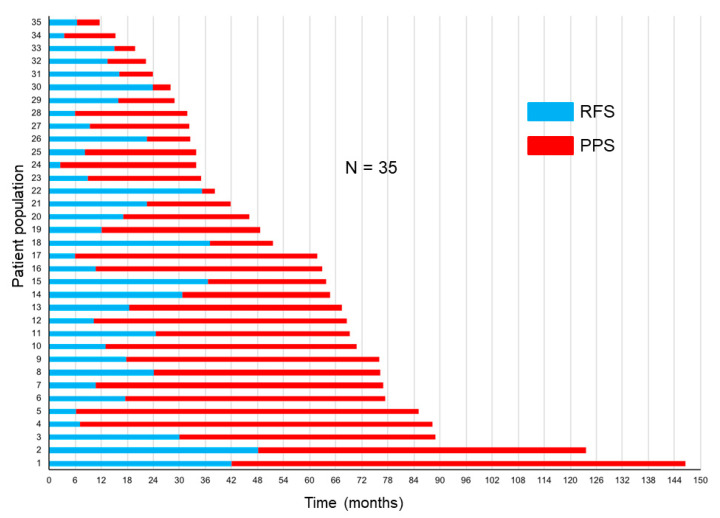
Relapse-free survival (RFS) and post-progression survival (PPS) in the entire study population.

The correlations of RFS and PPS with OS are demonstrated in Figure 3a,b. The Spearman’s rank correlation coefficients and linear regression revealed that the PPS strongly correlated with OS (*r* = 0.91, *p* < 0.0001, *R*^2^ = 0.85), whereas the RFS did not (*r* = 0.36, *p* = 0.03, *R*^2^ = 0.25). Figure 4 demonstrates the RFS and PPS of the entire study population.

### 3.3. Clinical Factors Affecting PPS

The univariate Cox regression analysis demonstrated that age at relapse, performance status (PS) at relapse, treatment with or without adjuvant chemotherapy, and presence of bone metastases at relapse were significantly associated with PPS (*p* < 0.05) (Table 3). In addition, the multivariate Cox regression analysis demonstrated that age at relapse and PS at relapse significantly influenced the PPS (*p* < 0.05, Table 3).

## 4. Discussion

We evaluated the correlations of OS with RFS and PPS at the individual patient level in patients who exhibited postoperative relapse of NSCLC harboring sensitizing *EGFR* mutations. The PPS was more significantly correlated with OS than RFS. Furthermore, age at relapse and PS at relapse were significantly associated with PPS. Most patients with postoperative relapse received regular follow-ups beyond surgical resection; therefore, the tumor burden might be lower than that in patients with metastatic NSCLC at initial diagnosis. These discrepancies in the burden and heterogeneity may favor PFS and OS in patients with postoperative relapse NSCLC [32,33,34].

Various trial endpoints have been examined in meta-analyses [35,36], and biostatisticians have suggested numerous assessment criteria to confirm alternative endpoints [37,38]. One study revealed that PPS, which was defined as survival post-progression, is significant for evaluating the adequacy of OS as a study endpoint [9]. PFS is generally defined as the survival time without “progression or death for any reason” after surgery. Relapse-free survival is generally defined as the survival time without “relapse or death for any reason” in the disease-free (cancer-free) state after a surgery. Other studies in patients with NSCLC have also demonstrated that PPS is highly correlated with OS beyond first-, second-, or third-line therapy [7,8,39]. Moreover, our previous studies revealed that patient-level data on PPS are relevant for evaluating early (first- and second-line) therapies in patients with advanced or metastatic NSCLC, as well as first-line treatment in patients with extensive-disease SCLC [10,40,41,42,43]. Therefore, in this study, we analyzed RFS and PPS in patients with postoperative relapse of *EGFR*-mutated NSCLC. Our findings demonstrated that RFS did not consistently correlate with OS, suggesting it may not be a valuable marker for prolonged OS. In addition, RFS was considerably shorter than PPS in the current cohort. PPS was highly associated with OS, suggesting that future clinical studies should consider the factors that may influence PPS.

A previous study of NSCLC has shown that prolonged PPS with first-line monotherapy and molecularly targeted drugs is strongly correlated with favorable PS [7]; though, the clinical factors influencing PPS at the individual patient-level in the postoperative relapse of NSCLC with sensitizing *EGFR* mutations remains unclear. In the current study, the multivariate analysis revealed that the following two factors were closely correlated with PPS: age at relapse and PS at relapse. This observation indicates that age at relapse and PS at relapse in patients with postoperative relapse of *EGFR*-mutated NSCLC may be important for prolonging PPS. The high number of anticancer agents administered beyond postoperative relapse can be attributed to the wide availability of first- and subsequent-line treatment options for NSCLC, including EGFR-TKIs (gefitinib, erlotinib, afatinib, and osimertinib), platinum-based combination regimen, pemetrexed, docetaxel, S1, and immune checkpoint inhibitors (ICIs) (Table 2). Osimertinib (a third-generation EGFR-TKI) demonstrated better drug-toxicity profiles than first- and second-generation EGFR-TKIs in trials, and their efficacies in patients with advanced or metastatic NSCLC with secondary T790M mutation and EGFR-TKI resistance are encouraging [20]. Although most cases in our cohort died before the evaluation of T790M mutation, if several patients with a secondary T790M *EGFR*-mutation are treated with osimertinib, the influence on PPS could be stronger than currently anticipated. Osimertinib use correlated with better PFS than current standard first-line therapies in patients harboring sensitizing *EGFR* mutations [19]. Therefore, osimertinib might be a more reliable standard front-line therapy for patients harboring sensitizing *EGFR* mutations. As first- and subsequent-line treatments are undergoing changes, the PPS beyond postoperative relapse in these patients might also show a change. Post-progression survival has a greater influence on the OS of patients with NSCLC harboring secondary T790M mutation when osimertinib is administered as a second-line therapy, in addition to first-line treatment with first- or second-generation EGFR-TKIs. However, PPS may be of value when using osimertinib as a first-line treatment after postoperative relapse. Current analyses imply that OS is more highly correlated with PPS than RFS in patients with *EGFR*-mutated NSCLC who underwent complete resection. Therefore, subsequent therapies might prolong the OS of these patients. The univariate analysis showed that the presence of a T790M mutation and the administration of osimertinib in the first-line treatment and ICIs were not statistically significant for PPS in the current analysis; however, this could be attributed to the small cohort scale.

The current analysis has some limitations. First, the cohort size was small. This limited our capacity to assess the relationships among PPS, the presence of T790M mutation, and the administration of osimertinib in first-line treatment and immune checkpoint inhibitors. Only a small number of postoperative relapse patients with *EGFR* mutations were available at our institution. Moreover, we tried to assess patients with the same backgrounds. Although a relatively large number of these patients were treated at our institution, our clinical practices and strategies are largely uniform. Adjusting for various sources of bias in the current analysis ensured clinically relevant results. Future studies with a higher number of patients are needed. Second, the treatment methods after postoperative relapse were not uniform, varying from drug therapy to radiotherapy alone. Nevertheless, this study, which is based on the actual clinical course of treatment, is of clinical relevance. Third, the date of response was determined by different treating physicians, and this may have resulted in a variability in the RFS; however, this is a limitation inherent to retrospective analyses. Fourth, we also managed to obtain censored survival data, although this does not affect the conclusions. Even when patients did not reach the death event, the RFS did not change. Besides, PPS and OS were prolonged, and PPS was closely correlated with OS.

In conclusion, PPS is more highly associated with OS than RFS in patients with postoperative relapse of *EGFR*-mutated NSCLC. Age at relapse and PS at relapse were also significantly associated with PPS. These outcomes imply that the course of treatment after postoperative relapse influences the OS of patients with *EGFR*-mutated NSCLC, though larger-scale prospective studies are needed to confirm these results in other clinical situations and patient cohorts.

## Figures and Tables

**Table 1:** Baseline characteristics of the patients.

Characteristic	*N* = 35
Sex	
Male/female	17/8
Median age at treatment (years)	69 (44–83)
Performance status (PS)	
0/1/2/≥3	20/11/3/1
Smoking history	
Yes/no/unknown	18/17/0
Histology	
Adenocarcinoma/others	35/0
Pathological stage at diagnosis	
I/II/III/IV	15/10/10/0
Operation	
Lobectomy/pneumonectomy	35/0
Mutation type	
exon 19 del/exon 21 *L858R*/*G719X*/Compound */ex 19 duplication	14/16/2/2/1
Adjuvant chemotherapy	
Yes/no	19/16
Treatment with EGFR-TKI	
Yes/no	30/5
Presence of a T790 mutation at recurrence	
Positive/negative or unknown	4/31
Rechallenge with a first- or second-generation EGFR-TKI	
Yes/no	3/32
Treatment with osimertinib	
Yes/no	3/32
Treatment with immune checkpoint inhibitors	
Yes/no	3/32
Recurrent pattern	
Local recurrence/distant metastasis	6/29
Intracranial metastases at recurrence	
Yes/no/unknown	9/26
Liver metastases at recurrence	
Yes/no/unknown	4/31
Bone metastases at recurrence	
Yes/no/unknown	12/23
Postoperative radiation after recurrence	
Yes/no	20/15
Number of therapies after postoperative relapse	
0/1/2/3/≥4	4/16/8/6/1
Median (range)	1 (0–7)

* L858R + S768I, G719S + S768I. EGFR-TKI, epidermal growth factor receptor-tyrosine kinase inhibitor.

**Table 2:** Treatments after postoperative relapse.

	First Line	Second Line	Third Line	≥Fourth Line	Total
Gefitinib	14	2	0	0	16
Erlotinib	3	1	0	0	4
Afatinib	5	1	0	0	6
Osimertinib	4	1	2	0	7
Platinum combination	1	3	1	0	5
Platinum combination + ICIs	0	1	0	0	1
Docetaxel	2	0	0	0	2
Pemetrexed	0	0	3	0	3
S-1	0	0	1	2	3
First- or second-generation EGFR-TKI rechallenge	-	3	0	0	3
Immune checkpoint inhibitors	0	0	1	1	2
Chemoradiotherapy	1	0	0	0	1
Definitive thoracic radiotherapy	4	0	0	0	4
Others (anticancer agents)	0	0	1	2	3
Best supportive care	1	-	-	-	-

ICI, immune checkpoint inhibitor; EGFR-TKI, epidermal growth factor receptor-tyrosine kinase inhibitor.

**Table 3:** Univariate and multivariate analyses of patient backgrounds for post-progression survival.

	Post-Progression Survival					
	Univariate Analysis			Multivariate Analysis		
Factor	Hazard Ratio	95% CI	*p*	Hazard Ratio	95% CI	*p*
Sex						
Male/female	0.86	0.37–2.00	0.73			
Pathological stage at diagnosis						
I/II–III	1.68	0.61–4.61	0.30			
Age at relapse	1.08	1.02–1.16	**<0.001**	1.09	1.03–1.17	**0.0018**
PS at relapse	2.64	1.53–4.44	**<0.001**	3.07	1.69–5.58	**0.0004**
*EGFR* mutation type						
Major mutation/minor mutation	2.78	0.91–12.1	0.07			
Adjuvant chemotherapy						
Yes/no	0.36	0.12–0.97	**0.044**			
Presence of T790 mutation						
Positive/negative or unknown	1.57	0.45–4.03	0.43			
Rechallenge with first- or second-generation EGFR-TKI						
Yes/no	1.16	0.81–4.18	0.84			
First-line treatment with osimertinib						
Yes/no	1.66	0.26–5.96	0.52			
Treatment with immune checkpoint inhibitors						
Yes/no	1.91	0.29–7.15	0.43			
Recurrent pattern						
Local recurrence/distant metastasis	0.46	0.16–1.13	0.09			
Intracranial metastases at relapse						
Yes/no/unknown	1.13	0.36–2.98	0.81			
Liver metastases at relapse						
Yes/no/unknown	4.33	0.92–15.7	0.06			
Bone metastases at relapse						
Yes/no/unknown	3.41	0.99–1.04	**0.009**			
Postoperative radiation after relapse						
Yes/no	1.11	0.48–2.68	0.79			
Number of therapies after postoperative relapse	0.86	0.56–1.23	0.46	□	□	□

Values in bold typeface were significant (*p* < 0.05). CI, confidence interval; PS, performance status; *EGFR*, epidermal growth factor receptor gene; EGFR-TKI, EGFR-tyrosine kinase inhibitor. *p*-values in bold are statistically significant (*p* < 0.05).

## Data Availability

The data presented in this study are available on request from the corresponding author. The data are not publicly available.
